# Anthrax Lethal Toxin and the Induction of CD4 T Cell Immunity

**DOI:** 10.3390/toxins4100878

**Published:** 2012-10-19

**Authors:** Stephanie Ascough, Rebecca J. Ingram, Daniel M. Altmann

**Affiliations:** 1 Section of Infectious Diseases & Immunity, Department of Medicine, Imperial College, Hammersmith Hospital, Du Cane Road, London, W12 0NN, UK; Email: s.ascough@imperial.ac.uk; 2 Centre for Infection and Immunity, Queen’s University Belfast, Health Sciences Building, 97 Lisburn Road, Belfast, BT9 7AE, UK; Email: b.ingram@qub.ac.uk

**Keywords:** anthrax, lethal factor, protective antigen, CD4 T cell, vaccine, epitope

## Abstract

*Bacillus anthracis* secretes exotoxins which act through several mechanisms including those that can subvert adaptive immunity with respect both to antigen presenting cell and T cell function. The combination of Protective Antigen (PA) and Lethal Factor (LF) forming Lethal Toxin (LT), acts within host cells to down-regulate the mitogen activated protein kinase (MAPK) signaling cascade. Until recently the MAPK kinases were the only known substrate for LT; over the past few years it has become evident that LT also cleaves Nlrp1, leading to inflammasome activation and macrophage death. The predicted downstream consequences of subverting these important cellular pathways are impaired antigen presentation and adaptive immunity. In contrast to this, recent work has indicated that robust memory T cell responses to *B. anthracis* antigens can be identified following natural anthrax infection. We discuss how LT affects the adaptive immune response and specifically the identification of *B. anthracis* epitopes that are both immunogenic and protective with the potential for inclusion in protein sub-unit based vaccines.

## 1. Introduction

Ever since Pasteur developed the first anthrax vaccine containing attenuated live organisms, clinical anthrax vaccines have largely been empirically derived [1,2,3]. There are two main types of anthrax vaccine available for use in humans; cell-free filtrates of antigenic proteins derived from cultures of avirulent *B. anthracis* strains and live attenuated spore-based vaccines. The live vaccines were developed from avirulent, non-encapsulated Sterne strains (ST-1 and ST-3) and predominantly used in Russia [2,4]. These vaccines may be administered by scarification of the skin, aerosol or subcutaneous injection, and require annual boosters [5,6]. Live vaccines have a low reported rate of adverse events [5], however, there are concerns regarding the use of live spores, as well as a dearth of data regarding the immunogenicity of these vaccines [6]. This means that currently, there are two anthrax vaccines widely used in humans, the UK licensed anthrax vaccine precipitated (AVP), and the US licensed anthrax vaccine adsorbed (AVA or Biothrax), both of which are based upon culture filtrates of *B. anthracis* [1,7,8]. The AVA vaccine is produced from a filtrate of the non-encapsulated, nonproteolytic strain V770-NP1-R, which is adsorbed onto aluminium hydroxide [9]. The UK vaccine is produced from an alum precipitated filtrate of the avirulent, non-encapsulated Sterne strain 34F2 [1,8]. Protective immunity is induced by administering the vaccines subcutaneously, in a series of up to six initial doses [7,8], followed by annual booster vaccinations. This extensive vaccination regimen, in combination with reported adverse reaction rates of 11% for the UK vaccine [10], and up to 60% for the US vaccine [8], has prompted the need for rationally designed, effective vaccines with low reactogenicity [1]. 

Both the AVA and AVP vaccines contain Protective Antigen (PA) and variable amounts of the two enzymatically active toxin subunits, Lethal Factor (LF) and Edema Factor (EF), which combine to form binary exotoxins [3]. In addition to their importance as key virulence factors for *B. anthracis*, these toxins form the basis of next generation anthrax vaccines currently under development. Human clinical trials have shown that vaccines based upon the protein sub-units are capable of eliciting T cell and antibody immune responses while avoiding the adverse reactions associated with older filtrate based vaccines [11,12,13]. 

As PA was the earliest focus of work to identify an immunologically protective antigen and remains the most extensively characterized of the anthrax toxins, it is perhaps unsurprising that the vast majority of approaches described to date have concentrated upon PA as the sole vaccine immunogen [14,15,16,17]. AVA and recombinant protective antigen (rPA) based vaccines have been found to induce anti-PA antibodies in a variety of animal species [18], antibodies which were also correlated with survival in animals challenged with anthrax infection [19], and could be passively transferred to naive animals [20]. In addition to anti-PA IgG, immunisation of rabbits and guinea pigs with AVA or rPA also induced the production of toxin neutralising antibodies specific to PA, levels of which were predictive of survival following anthrax infection [21,22,23,24,25]. This indicated that the presence of toxin neutralising antibodies, which could also be passively transferred to naive animals [25], conferred protective immunity to anthrax. Furthermore, Weiss *et al.* discovered that in rabbits immunised with PA based vaccines the toxin neutralising antibody titres were better predictors of survival following anthrax challenge than total anti-PA antibody titres [26].

Despite this, human and animal vaccination studies have indicated that not only PA, but also LF is capable of conferring protective immunity [3,27,28]. Human vaccinees, following immunisation with either the AVA or AVP vaccines, show antibody responses to both PA and LF [29,30], while both PA and LF specific antibodies have been detected in sera taken from naturally infected anthrax patients [29,31]. A case study involving a naturally acquired cutaneous anthrax infection notably demonstrated detectable anti-LF antibodies in the absence of anti-PA antibodies [32]. This was supported by the recent work by Brenneman *et al.* which found that in cutaneous anthrax patients, the majority of toxin specific IgG antibodies were directed against LF, and were induced considerably earlier than either PA or EF specific antibodies [33]. Animal studies have found that one year after PA based vaccinations there was minimal protection against infection [18,34], providing evidence that PA based immunity may not be long lasting. Crowe *et al.* also recently discovered that while the overwhelming majority of AVA vaccinated humans in a study cohort demonstrated PA-specific antibody responses, many of the responses were not actually associated with a detectable toxin neutralising effect [35]. As a link has been established between the passive protection afforded to mice against both toxin challenge [36,37] and anthrax infection [38], by toxin neutralising monoclonal antibodies generated from AVA vaccines, it suggests that current vaccines, such as AVA, which focus upon induction of anti-PA antibody responses, may produce a variable quality of immunoprotection. Antibodies directed against LF have proven to be toxin neutralising, both *in vitro* and *in vivo* in rats [39] and mice [36]. The growing body of work on LF, identifying B cell epitopes [40,41], providing evidence that LF can boost the magnitude of PA-specific antibody responses in mice following co-administration [42,43], and that LF truncate proteins confer protection against *B. anthracis* aerosol challenge [17,44], adds weight to the evidence that responses against LF may be more important mediators of protective immunity than previously thought, with the potential for inclusion of LF derived epitopes in a sub-unit vaccine. 

In contrast to the focus on antibody-based immunity which has driven studies of *B. anthracis* antigens to date, there is a paucity of work on T cell immunity, with those few studies which exist mainly concentrating on responses to PA [45,46,47]. The discovery that guinea pigs which survived *B. anthracis* exposure exhibited extremely low anti-PA and LF antibody titres indicated that protection was not necessarily associated with the induction of antibody responses [29]. However, it is only recently that the critical role cellular immunity plays in the clearance of *B. anthracis* has been investigated more fully, questioning the primary importance of humoral immunity in providing protection against anthrax infection [48]. Our own recent research has shown that LF specific IFNγ producing CD4+ T cells play an important role in generating long lasting immunity to anthrax [49]. In individuals exposed to anthrax spores, circulating T cells reactive to both LF and PA have been identified [50], and low-level anthrax exposure has been found to lead to PA and LF specific cellular responses in the absence of detectable antibodies [51]. The importance of considering the cellular immune response to both the PA and LF immunogens echoes a growing trend in the vaccinology field that a focus upon eliciting antibody responses should not be the sole approach for pathogens that encompass an intracellular component to their pathogenesis [52]. It is thus crucial that we understand how these exotoxins impact on adaptive immunity, as well as their potential in eliciting more effective T cell immunity. 

## 2. The *B. anthracis* Lethal Factor Toxin

The entry of *B. anthracis* exotoxins into host cells is mediated by PA, which binds to two cell surface receptors; anthrax toxin receptor 1 (ANTXR1), also known as tumour endothelial marker 8 (TEM8) and anthrax toxin receptor 2 (ANTXR2), also known as capillary morphogenesis gene 2 (CMG2) [53,54,55,56]. Both of these receptor proteins, whose normal physiological function in the host concerns capillary development, bind to PA through the cation-dependent metal ion-dependent adhesion site (MIDAS), within their von Willebrand A (vWA) domains, which are also referred to as “integrin-like” domains [57,58]. Cohen *et al.* have recently demonstrated *in vitro* that α4β1- and α5β1-integrin complexes are capable of binding to PA and mediating transport of the anthrax toxins into the cell [59]. Whilst the discovery of a third PA receptor on the host cell may prove to have interesting implications for efforts to develop monoclonal antibodies, blocking PA binding to host cell receptors, currently ANTXR2/CMG2 remains the most physiologically relevant receptor during anthrax infection. Not only did Cohen *et al.* establish that the integrin receptors bound *in vitro* to PA with an affinity comparable to that of ANTXR1/TEM8, much lower than ANTXR2/CMG2 [59], but also the *in vivo* toxicity of anthrax toxin is predominantly mediated by ANTXR2/CMG2 rather than ANTXR1/TEM8, due to both the high affinity PA displays for the ANTXR2/CMG2 receptor and the higher levels of this receptor expressed upon cell populations within the host [60,61,62]. PA forms heptameric structures to which the EF and LF proteins bind through a homologous *N*-terminus region, forming Edema Toxin (ET), or Lethal Toxin (LT), which are transported into the cytosol. The structure and function of PA and EF have been extensively reviewed elsewhere; in this issue we will concentrate primarily on LF and its mode of action.

### 2.1. The Structural Domains of LF

**Figure 1:**
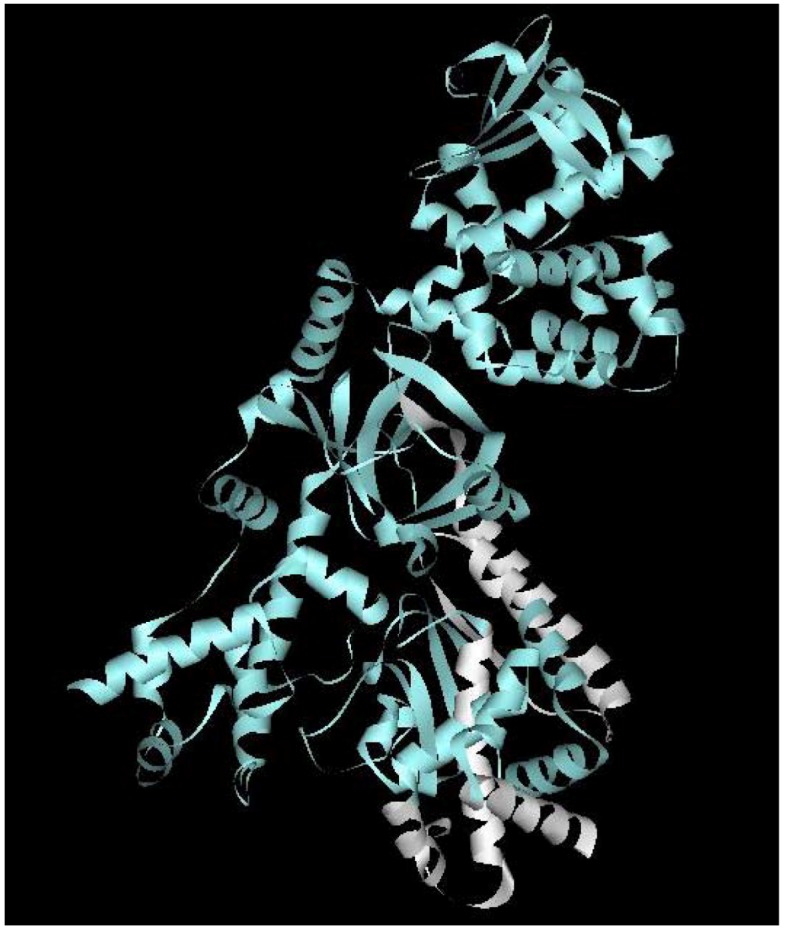
A schematic ribbon diagram depicting the Lethal Factor protein. Within the domain IV catalytic center we have identified a number of T cell epitopes, represented in white [49]. LF is capable of inactivating key cellular pathways, as the majority of these functions are related to the active center, epitopes within this region may prove crucial in the development of a vaccine capable of successfully inhibiting the toxin. This figure was generated using the Accelrys discovery studio client 2.5 program.

LF is a zinc dependent metalloprotease, composed of four domains following a predominantly helical structure [63] (Figure 1). Following the AAAGGAG ribosome-binding sequence, the upstream ATG start codon begins a continuous 3631 bp open reading frame which encodes the 809 amino acids composing the LF precursor protein (Gen Bank accession number M30210) [64,65]. The first 99 bp of the open reading frame encodes a 33 amino acid signal peptide, which is homologous to leader peptides for other proteins secreted by members of the *Bacillus* genus [65]. The first 300 amino acids of the α-helical domain I, of the 776 amino acid long mature LF protein, share extensive homology with that of the EF protein [66]. In both EF and LF, these amino terminal regions are critical for binding to the amino terminus subdomain I of the PA protein [66]. Analysis of the LF binding motif confirms that only the first 254 amino acids of LF are required for PA binding and translocation into host cells [67]. This has been exploited by groups who found that vaccine candidates fused to truncated subdomains of LF were efficiently delivered to the cell cytosol and were thus capable of eliciting a cell mediated immune response [67,68].

The helix bundle making up domain III is inserted into domain II, and is believed to have arisen from a series of imperfect repeated duplications of a structural element comprising domain II [69]. Domain III is characterized by the presence of the four repeats of the amino acids 293 to 300 of domain II, deletions in this region abrogated the catalytic activity of LF, indicating that this region is essential to the stability and function of the LF catalytic site [70]. Domain II itself resembles the adenosine diphosphate (ADP)-ribosylating toxin, found in the *Bacillus cereus* vegetative insecticidal toxin 2 (VIP2), although its active site has undergone mutation [63]. Mutagenesis studies indicate that several domain II residues; Leu^253^, Lys^254^, Arg^491^, Leu^514^ and Asn^516^ are involved in substrate recognition and binding [71]. Along with domain III, the domain II protein appears to have become involved in substrate recognition and binding [63]. 

Domain IV contains the zinc protease active site [72]. This domain is predominantly α-helical, with the exception of a 4-stranded β sheet, which is a characteristic of other zinc proteases [63,72]. Analysis of the carboxyl terminus amino acid sequence revealed that the amino acid residues 686 to 692 (HEFGHAV) contained a classical binding motif conserved across the thermolysin-like metalloproteases [72]. The active site lies within a deep cleft formed by domains II, III and IV. A zinc ion is coordinated tetrahedrally to domain IV by three of the active site residues; Glu^687^ which acts as a ligand for a zinc bound catalytic water molecule, and the residues His^686^ and His^690^, which directly coordinate the zinc ion itself [63,73]. Tonello *et al.* also suggested a role for Glu^735^, which aids in the co-ordination of the zinc ion [74]. 

While the majority of the active site residues share similarity with the thermolysin family of metalloproteases, an essential tyrosine residue has also been identified which is absent in the thermolysins. The domain IV residue Tyr^728^ has been implicated in catalysis, acting to protonate the departing amino terminal of the active site substrate [63,74]. The Tyr^728^ position in LF shows similarity to the essential Tyrosine residue present in the clostridial metalloprotease neurotoxins, specifically the Tyr^366^ found in *Clostridium botulinum* neurotoxin type A and the Tyr^375^ found in *Clostridium tetani* tetanus neurotoxin [74,75,76]. The phenolic OH group of the domain IV Tyr^728^ residue, in addition to the conserved thermolysin-like binding motif appears to be critical to the catalytic action of LF upon the Mitogen-Activated Protein Kinase Kinase (MAPKK) host substrates.

### 2.2. The Action of LT upon the MAPK Signaling Cascade

**Figure 2:**
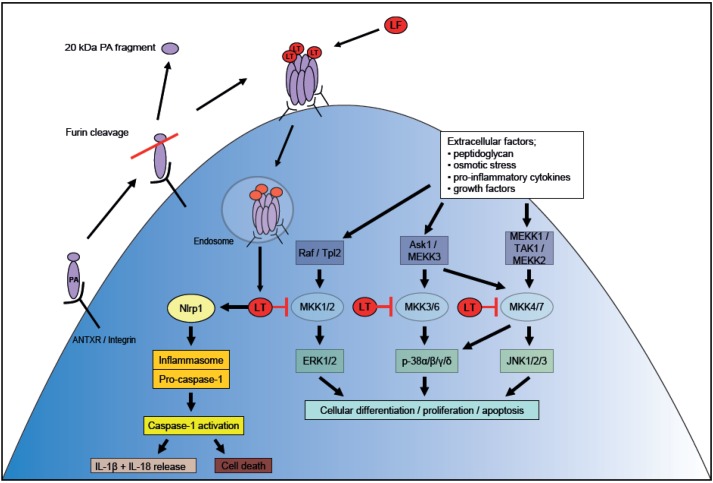
Model of cellular intoxication by LT. The 83 kDa PA protein secreted by *B. anthracis* binds to the host cell surface receptor (ANTXR1/TEM8, ANTXR2/CMG2 or the integrins α4β1 and α5β1), and is proteolytically cleaved by host furin, releasing a 20 kDa fragment from the *N*-terminal of the protein. The remaining 63 kDa PA fragment heptamerises and binds to LF to form LT. The toxins are then internalised in endosomal vesicles which are subsequently acidified, triggering the translocation of LT into the host cytosol. LT inactivates the MKKs, the central step in the MAPK signaling pathway, and induces the Nlrp1 activation of the inflammasome.

Within the cell, the catalytically active *C* terminal domain IV of LT engages in a highly specific proteolytic cleavage of the amino terminal extension of MAPKKs or MKK family members [77]. MKKs are the central components in a three stage signaling cascade which is initially activated when the cell is stimulated by extracellular factors such as the peptidoglycan found in the cell wall of *B. anthracis*, osmotic stress, pro-inflammatory cytokines and growth factors [78] (Figure 2). This induces activation of the Mitogen-Activated Protein Kinase Kinase Kinases (MKKK or MEKK) family members. The most well characterised members of this family are MEKK1 and Raf [72,79], although members include; MEKK2, MEKK3, MEKK4 [80,81], tumor progression locus 2 (Tpl2) [82], apoptosis signal regulating kinase 1 (Ask1) [83] and TGFβ activated kinase (TAK1) [84]. The MEKK family members then phosphorylate the serine and threonine residues of MKKs; once activated these dual-specificity kinases are then responsible for the reversible dephosphorylation of both the serine and tyrosine residues on MAPK [85]. 

LT acts to disrupt the activity of three of the four families of MAPKs [86]; Extracellular Signal Regulated Kinases (ERK1 and 2, which are activated by MKKs 1 and 2, and are responsible for regulating cellular differentiation and proliferation) [78], c-Jun *N*-terminal kinases (JNK1, 2 and 3, which are activated by MKKs 4 and 7, and are known as Stress Activated Protein Kinases) [78], and the p38 isoforms (α, β, γ and δ which are activated by MKKs 3, 4 and 7 and are involved in regulating cell differentiation and apoptosis) [85]. Only the ERK5 (activated by MKK 5) signaling transduction pathway is not disrupted by the activity of LT within the cell [64]. 

LT preferentially cleaves proline residues [73]. Alignment of the amino acids which comprise the NH_2_ terminal of the MKK families, demonstrate that they show a limited homology within the first 20 amino acids, centering around two proline residues, separated by one or two amino acids and preceded by a sequence of basic residues [64,87,88]. Cleavage at these conserved proline rich regions, which precede the catalytic kinase domains, disrupts or removes a D-site (also known as a DEJL motif or D-domain), which is a protein interaction site, essential for high affinity binding to MAPKs [87]. LT cleaves the D-sites of MKK1, MKK2, MKK3 and MKK6 once, while MKK4 is cleaved twice and MKK7 contains three putative cleavage sites [87,89]. Only the action of MKK5 is not disrupted by LT cleavage, the reason for this may lie in the lack of homology that the hydrophobic *N*-terminal domain of MKK5 shows to the other MKK D-sites; the MKK5 MAPK binding region instead consists of a stretch of negatively charged residues which are resistant to LT cleavage [89,90]. These MKK D-sites therefore appear to represent a major target of LT substrate recognition and catalysis, and disruption of these regions interferes with MKK binding interaction to MAPK, causing the LT mediated inhibition of the MAPK signaling pathway [87]. 

### 2.3. Involvement of LF in Caspase-1 Activation

The research by Friedlander *et al.* was the first to establish the cytotoxic effect LT has upon host immune cells, preferentially targeting activated mouse macrophages [91]. Further research has revealed that not only macrophages, but also murine DCs and B cells, are more sensitive to LT intoxication than T cells, with both murine CD4+ and CD8+ cells proving to be relatively resistant to LT induced cell lysis [62]. The toxin sensitivity specific to the phagocytic APCs was expected to impact upon the presentation of antigens from *B. anthracis*, allowing the bacteria to evade both the innate and adaptive immune response [62,92]. However, an inverse relationship exists between toxin sensitivity and resistance to infection, with several studies demonstrating that animals with APCs sensitive to toxin induced lysis are resistant to challenge with *B. anthracis* spores [93,94,95,96]. Furthermore, the mechanism of LT dependant APC death was found to be unrelated to its proteolytic effects upon the MAPK signaling cascade, as both toxin resistant and sensitive macrophages showed equal sensitivity to MKK cleavage [97,98], indicating that this effect was instead related to a previously unknown function of LT. 

The genetic basis of the differential susceptibility to LT in mice was initially mapped to a locus in chromosome 11 [99,100], leading to the discovery that polymorphisms in the gene *Nlrp1b* govern the control of macrophage and DC sensitivity to LT [101]. The *nlrp* genes encode a family of pattern recognition receptors (PRR), known as nucleotide oligomerization domain-like receptors (NLRs), which allow innate immune cells to recognize and respond to pathogen associated molecular patterns. The NLR family forms the multi-protein inflammasome complex. This scaffold protein oligomerizes to recruit and activate caspase-1, driving pro-inflammatory cell death, known as pyroptosis, which is associated with lysosomal membrane permeabilization and the release of cathepsins into the cell cytosol [58]. The related caspase-1 directed cleavage of the pre-cursor forms of IL-1β and IL-18, which are then released as mature cytokines, also helps drive the inflammatory response [102,103] (Figure 2). The expression of a resistant or sensitive allele (*Nlrp1b^R^* or *Nlrp1b^S^*) governs the mouse macrophage response to LT intoxication, and consequently resistance to anthrax infection. In mice which possess the *Nlrp1b^S^* gene, the inflamasome-mediated cell lysis promotes a pro-inflammatory response predominantly driven by IL-1β, which recruits immune cells to the site of LT exposure to resolve infection [102,104]. Knocking out the IL-1 receptor and caspase-1 in *Nlrp1b^S^* mice negates any resistance to spore challenge conferred by this allele, suggesting that it is these immune mediators which are primarily responsible for protection from infection seen in *Nlrp1b^S^* mouse strains [103,104]. Furthermore, macrophages from caspase-1 deficient mice displayed an impaired ability to kill *B. anthracis in vitro*, which was restored by addition of rIL-1β, but not rIL-18 [105], suggesting that IL-1β is the critical product of caspase-1 in the context of the host response to *B. anthracis*. 

Although the recent work by Terra *et al.* [104] does indicate that the allelic variations in *Nlrp1b* do not fully account for the responses of different strains to infection and LT intoxication. They hypothesise that one or more, as yet, unidentified additional genes, within the quantitative trait loci (QTL) of mouse chromosome 11 known as *Lethal toxin sensitivity 1* and *2* (*Ltxs1* and *Ltxs2*), may play a role in the induction of an inflammatory host response to, not only LT, but also stimuli such as lipopolysaccharide (LPS) [99,104,106]. *Nlrp1b* itself resides within *Ltxs1* [101], and there is a possibility that analysis of this critical region may reveal further genes which influence the course of bacterial diseases, such as sepsis, with a strong link to the release of pro-inflammatory cytokines by the host. A different interpretation has been offered by Kang *et al.*, who found that IL-1β expression was induced by both the non-germinating *B. anthracis* spores and the LT produced by vegetative bacteria [105]. The spores induce the cooperative regulation of IL-1β expression by both the MyD88-dependent induction of pro-IL-1β and the MyD88-independent pathway involving the IFN-β induced STAT1 activation, which is required for the caspase-1 processing of pro-IL-1β to the mature cytokine [105]. This mirrors the role of IFN-β in the activation of the inflamasome during the host response to other bacterial species, such as *Francisella tularensis* [107]. Low doses of *B. anthracis* spores both *in vitro* and *in vivo* induced caspase-1 activity and IL-1β mRNA and protein production, but low levels of cell death, in contrast to which, exposure to LT induced the production of the IL-1β protein, and increased cell death, but was associated with low levels of IL-1β mRNA expression [105]. It has been speculated that LT acts to liberate pre-formed IL-1β from lysed cells [108], and Kang *et al.* found that *B. anthracis* spores induced the expression of IL-1β mRNA and protein without macrophage cell death in strains of mice reported to be both susceptible and resistant to LT intoxication [105]. They suggest that the activation of caspase-1 by anthrax spores therefore requires a different intracellular receptor to the Nalp1b required by LT, highlighting that the caspase-1 activation induced by *F. tularensis* utilises another member of the NLR family, the adaptor protein ASC [107]. It is therefore possible that the *B. anthracis* spores and the LT may present distinct agonists which engage different signaling pathways at different timepoints during infection to induce the production of IL-1β. 

Further evidence for the importance of the inflammatory mediators is provided by the rat ortholog *Nlrp1*, found on chromosome 10, which produces an Nlrp1 protein cleaved by LT within an *N*-terminal domain [109,110]. The Nlrp1 directed activation of the inflammasome and the subsequent caspase-1 dependent macrophage death, is subject to the same polymorphisms which occur in Nlrp1b, leading to the different susceptibilities to LT intoxication between rat strains [109,110,111]. In contrast to the murine strains, however, *Nlrp1^S^* mediated control of anthrax infection does not occur, instead susceptibility to infection mirrors macrophage susceptibility to toxin induced cell death [110]. Newman et al have suggested that whilst exposure to LT activates inflammasome assembly and caspase-1 activity in *Nlrp1^S^* rats, this occurs 30–45 min later than in *Nlrp1b^S^* mice, furthermore, they also found that systemic levels of IL-1β were lower in *Nlrp1^S^* rats [111].

It is intriguing that the polymorphisms governing susceptibility or resistance to LT dependent cell death have been found to lie within the *N*-terminal domain of the rodent Nlrp1 paralogs. This region, which contains the recently identified LT cleavage site, differs substantially from corresponding human NLRs, which contain a pyrin domain responsible for binding to the ASC inflammasome adaptor protein, required for IL-1β processing [112]. The limited sequence homology between the murine and human *N*-terminal domains, resulting in an absent cleavage site, has been cited as a contributing factor in the resistance to LT intoxication which has been observed in all human macrophages tested to date [111]. If true, this would predict that humans react to anthrax infection in the same manner as *Nlrp1b^R^* mouse strains, however the functional, long-term, humoral and cellular immunological memory identified in survivors of clinical anthrax infections indicates that humans are capable of mounting a robust immune response [49,113]. We have recently found that cells from individuals with cutaneous anthrax infections produce high levels of IL-1β following challenge with LF antigens [114]. This pro-inflammatory cytokine has proven crucial to the survival of *Nlrp1b^S^* mice during anthrax infection [104], and it is tempting to speculate that although an LF cleavage site has not yet been identified within the human Nlrp1 protein, a corresponding pathway for caspase-1 activation and cytokine release protects human macrophages. 

## 3. LT and T Cell Immunity

In contrast to the LT induced activation of caspase-1, which can take up to 60 min [115], MKK cleavage is extremely rapid, occurring within 20 min of LT addition to cells *in vitro* [116]. The MAPK cascades are critical in controlling T-cell activation, as these pathways are involved in the activation of T cell receptor (TCR) dependent nuclear factor of activated T cells (NFAT) family members. Along with activating transcription factor 2 (ATF2), the NFAT family members act synergistically with nuclear subunit activation protein 1 (AP-1) as transcription factors regulating the expression of inducible genes vital to the adaptive immune response. The regulation of NFAT, AFT2 and AP-1 activation is controlled at multiple levels by MAPK phosphorylation, the action of LT upon the MKKs thus blocks JNK, ERK and p38 mediated T cell proliferation [117,118]. The NFAT transcription factors are also involved in the regulation of genes encoding IL-2 and the IL-2 Receptor (IL-2R), which are essential for the clonal expansion of antigen specific T cells. LT induced impairment of T cell proliferation is traditionally associated with the reduced production of the Th1 and Th2 cytokines IL-2, IFNγ, TNFα and IL-5 and the downregulation of the activation markers, CD69 and CD25 [117,119]. The blockade of T cell activation being potent enough to inhibit the co-stimulation *ex vivo* of LF exposed CD4+ T cells, by α-CD3 and α-CD28 antibodies [118]. Due to its role in the perturbation of PKA dependent intracellular signalling pathways, EF also has an indirect effect upon NFAT transcriptional regulation, interfering with the calcium dependent signalling, but only when present at much higher concentrations than LF [117]. 

The antagonistic effect of EF upon PKA strengthens the effects of LF on MAPK cascades, as EF is capable of both phosphorylating a negative regulatory residue of Raf, which lies upstream of the MAPK pathway, and phosphorylating a positive regulatory residue of Rap1 which inhibits Ras initiation of the MAPK cascade [120]. The synergistic effect of LF and EF upon the MAPK pathways suppresses T cell chemotaxis in response to CXCL12 [120]. This may be mediated through inhibiting the phosphorylation of both regulatory components such as paxillin and structural components such as actin, of the cell cytoskeleton [121,122]. The elevation of intracellular cAMP by EF also appears to skew the differentiation of naïve CD4+ T cells towards a Th2 subset, inhibiting the TCR dependent activation of Akt1, a protein essential for the development of a Th1 subset, whilst enhancing the activation of the guanine nucleotide exchanger Vav1 and the stress kinase p38 which are involved in Th2 differentiation [123]. A predominantly Th2 response has also been reported following stimulation of PA-specific T cells from AVA immunised rhesus macaques typified by increased levels of IL-4 and IL-2 but not IFNγ, IL-6, IL-1β or TNFα mRNA levels [18]. Boyaka *et al.* have also found that PA specific CD4+ T cells from mice immununised with rPA secrete low levels of Th1 associated cytokines, but increased levels of characteristic Th2 cytokines IL-4, IL-5 and IL-10 [124]. It is important to note however, that both studies utilised adjuvants which may have had an impact upon the induction of distinct T cell subpopulations, as both the alum adjuvant in AVA vaccines and the cholera toxin adjuvant used by Boyaka *et al.* have been implicated in biasing the CD4 T cell response towards a Th2 population [125,126,127]. Along with the LF and EF effect upon DC cytokine release, which promotes preferential Th2 differentiation, this represents both direct and indirect consequences of anthrax toxins upon the lineage commitment of naïve CD4+ T cells. 

The importance of an IFNγ CD4+ T cell response in mediating protection from anthrax infection *in vivo* [48], in addition to the increased survival and *B. anthracis* bactericidal activity of IFNγ activated macrophages [128], and the disproportionate focus of LT upon suppressing IFNγ release by NK cells [129], suggests that survival of *B. anthracis* within the host depends in part, upon the suppression of a Th1 associated IFNγ release. Conversely this also indicates that the induction of an IFNγ producing memory T cell population should be an important goal of any future *B. anthracis* vaccine design.

Recent research has suggested a role for natural killer (NK) cells in the production of IFNγ following exposure to anthrax spores [130], and natural killer T (NKT) cells in the generation of PA-specific, LT neutralizing antibodies by B cells [131]. NKT-derived IL-4 and IFNγ influences the production of polyclonal PA-specific IgG1, which Devera *et al.* found to be more protective *in vivo* than polyclonal PA-specific IgG2b or IgG2c [132]. However, it must be noted that Abboud *et al.* found that PA-specific IgG2a isotypes were superior to both IgG2b and IgG1 in terms of *in vivo* protection [133]. Devera *et al.* acknowledge that due to the absence of IgG2a isotypes within their work, IgG1 may have assumed a greater importance than it might otherwise warrant, although the passive transfer of monoclonal antibodies in the model of Abboud *et al.* could equally have led to a greater role for IgG2a than may yet be demonstrated within a natural anthrax infection. 

The CD1d restricted type I invariant NKT (iNKT) cells provide B cell help, in addition to the production of the pro-inflammatory cytokines IFNγ, TNFα and GM-CSF. Surprisingly iNKT cells appear to express higher levels of the PA receptors TEM-8/ANTRX1 and CMG-2/ANTRX1 than other hematopoietic cells [134]. As NKT cells represent less than 1% of the circulating T lymphocytes in humans (although this percentage varies substantially between individuals) this may denote a disproportionate focus upon this cell population by anthrax toxins. This preferential targeting of iNKT cells is supported by the downregulation of the activating receptor NKG2D on iNKT cells but not NK cells in the presence of LT [134]. LT is capable of mediating a variety of effects upon iNKT cells through the cleavage and inactivation of MKK2 which impacts upon the phosphorylation of downstream ERK1 and 2 [134,135]. Inhibition of the ERK pathway impairs CD1d mediated antigen presentation to iNKT cells, in addition to an observed reduction in endosomal trafficking of antigen loaded MHC class II molecules; this has major implications for the induction of both NKT and CD4+ Th1 adaptive immune responses to *B. anthracis* [92,135].

The inhibitory effects of both LT and ET upon expression of the activation markers CD25 and CD69 and the secretion of the pro-inflamatory cytokines IL-2, TNFα, and IFNγ by human T cells has been described *in vitro* [117,119]. Murine lymphocytes have also demonstrated impaired TCR mediated cell activation and CD4+ T cell production of the cytokines IL-3, IL-4, IL-5, IL-6, IL-10, IL-17, TNFα, IFNγ and GM-CSF following exposure to LT and ET [118]. However, the cellular immunity identified within naturally infected humans indicates that, although *in vitro* exposure to ET has been implicated in immune deviation towards both the Th2 and Th17 pathways [123,136], the human immune response against LF encompasses a broad range of cytokines associated with Th1, Th2, Th9 and Th17 subsets, indicating little or no helper T cell polarization [114]. This lack of skewing following repeated exposure to LF antigens echoes the work of the Peakman lab, who found that there was an equal balance of Th1 and Th2 responder cells in the recall immunity to anthrax [137]. Martchencko *et al.* [138] observed that anthrax toxin sensitivity, which varies between cell lines generated from different humans, appears to be strongly correlated with variation in the expression of the host cell surface receptor ANTXR2/CMG2. A single-nucleotide polymorphism (SNP), affecting the uptake of anthrax toxins, was identified in the protein coding region of ANTXR2/CMG2. Interestingly, the polymorphism, which is commonly found in African and European populations, decreased the cellular entry and subsequent lethality of anthrax toxin in murine cells, although this was not translated into a statistically significant difference between the *in vitro* human cell populations, in terms of toxin sensitivity. It remains unclear, at present, what role the genetic diversity of the host plays in the course of a *B. anthracis* infection, however two recent large scale studies have indicated differences in the generation of toxin specific antibodies between AVA vaccinees of different ethnicities. Crowe *et al.* reported that African American individuals displayed lower titres of toxin neutralising antibodies following vaccination [36], while Marano *et al.* found that, during the immunisation schedule, anti-PA IgG levels were significantly lower in African Americans when compared to Americans of European descent (although this effect was not observed at a time point seven months post-vaccination) [139]. As Ingram and Baillie observed, a range of genetic factors may govern this variation in response to anthrax derived proteins, including the expression of cellular receptors like ANTXR2/CMG2, polymorphisms in both cytokine and cytokine receptor genes, and variations in Toll like receptor (TLR) genes or HLA haplotype [140]. 

Extensive allelic polymorphisms observed within the HLA region influence the repertoire of antigenic epitopes presented by MHC class II molecules to CD4 T cells; in order to fully elucidate the cellular immune response to anthrax it is therefore important to define these epitopes. Most vaccine strategies against anthrax have concentrated upon PA, however LF has been identified as a major target of T cell immunity in humans [49]. As the amount of LF released by *B. anthracis* is one-sixth of that of PA [141], these findings indicate that LF may contain proportionally more epitopes which are of relevance in the T cell immune response. The elicited human T cell responses indicated that the key LF epitopes, LF_574–593_, LF_654–673_, LF_674–693_ and LF_714–733_, are concentrated in domain IV (Figure 1). It is interesting to note that this reveals a very different pattern to that of the B cell epitope mapping performed by Nguyen *et al.*, who found that the majority of structural B cell epitopes clustered in domains I and III of LF [41]. 

The binding groove created by domains II, III and the catalytically active center of the LF toxin, domain IV, is responsible for holding the 16 amino acid long peptide which makes up the tail of the MAPK kinase family [63], and it has been observed that mutations in the sequence coding for domain IV eliminates the peptidase activity of LF, abrogating its toxicity [142]. The putative zinc binding site, which lies between the amino acid residues LF_686_ and LF_692_ [73,77] was a feature of the response to LF_674–693_; these represent important residues for zinc binding and catalysis as well as cytotoxicity, which are conserved across this class of metalloprotease [72]. The immunodominant epitopes identified within domain IV therefore appear to comprise essential residues of LF which are critical for efficient catalytic activities and the execution of substrate cleavage. 

## 4. Conclusions

The cleavage of MAPK kinases by LT inhibits the evolutionarily conserved MAPK signal transduction pathway and has multiple downstream consequences for both the innate and adaptive immune systems. The resulting immune subversion might be expected to impact upon the T cell memory of *B. anthracis*, however it is clear that the involvement of factors such as the Nlrp1 directed inflammasome allow the immune system to develop antigen-specific responses to LF. We propose that in addition to resolving infection, the cell infiltration and cytokine milieu seen in early inflammation are crucial in driving antigen presentation and T cell priming which results in broad Th cell subset responses to LF, which can be detected several years after the initial infectious event. The identification of T cell epitopes from the LF protein may therefore be beneficial in developing a future protective sub-unit based vaccine.
